# Supplementary material for: ZnO Nanowires on Single-Crystalline Aluminum Film Coupled with an Insulating WO3 Interlayer Manifesting Low Threshold SPP Laser Operation
Source: Nanomaterials (Basel). 2020 Aug 27;10(9):1680. doi: 10.3390/nano10091680 (PMC7557600; doi:10.3390/nano10091680)
Supplement: Supplementary file 1 [file nanomaterials-10-01680-s001.pdf]

nanomaterials

Supplementary Materials

ZnO Nanowires on Single-Crystalline Aluminum Film Coupled with an Insulating WO_3 Interlayer Manifesting Low Threshold SPP Laser Operation

Aanchal Agarwal [1], Wei-Yang Tien [1], Yu-Sheng Huang [1], Ragini Mishra [2], Chang-Wei Cheng [3], Shangjr Gwo [3,4], Ming-Yen Lu [1,*], and Lih-Juann Chen [1,4,*]

[1] Department of Material Science and Engineering, National Tsing Hua University, Hsinchu 30013, Taiwan, agarwal.aanchal@outlook.com (A.A.); young951700@gmail.com (W.T.); samxgoodguy@hotmail.com (Y.H.);
[2] Institute of NanoEngineering and MicroSystems, National Tsing Hua University, Hsinchu 30013, Taiwan, argn.mishra30@gmail.com;
[3] Department of Physics, National Tsing Hua University, Hsinchu 30013, Taiwan, pir820314@gmail.com (C.C.); gwo@phys.nthu.edu.tw (S.G.)
[4] Frontier Research Center on Fundamental and Applied Sciences of Matters, National Tsing Hua University, Hsinchu 30013, Taiwan
* Correspondence: ljchen@mx.nthu.edu.tw (L.C.); mylu@mx.nthu.edu.tw (M.L.)

1. Schematic representation of a ZnO nanowire placed on a WO_3 dielectric layer coated Al film

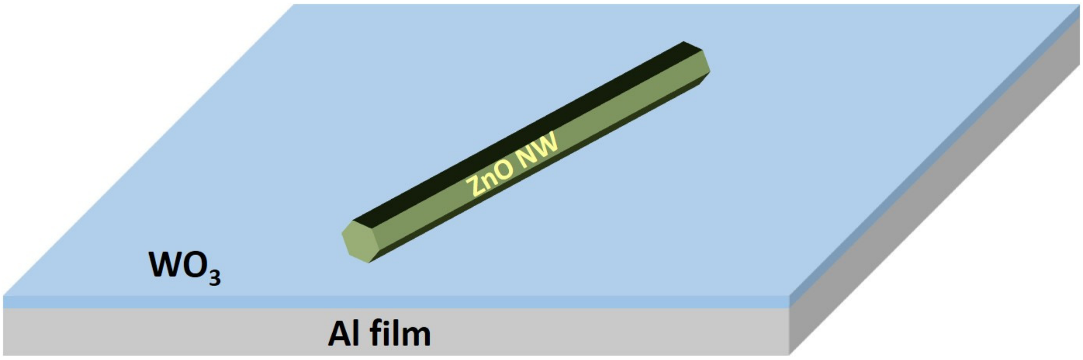

Figure 1. Schematic representation of a ZnO nanowire placed on a dielectric layer coated Al film.

2. Growth process setup for synthesis of crystalline ZnO nanowires

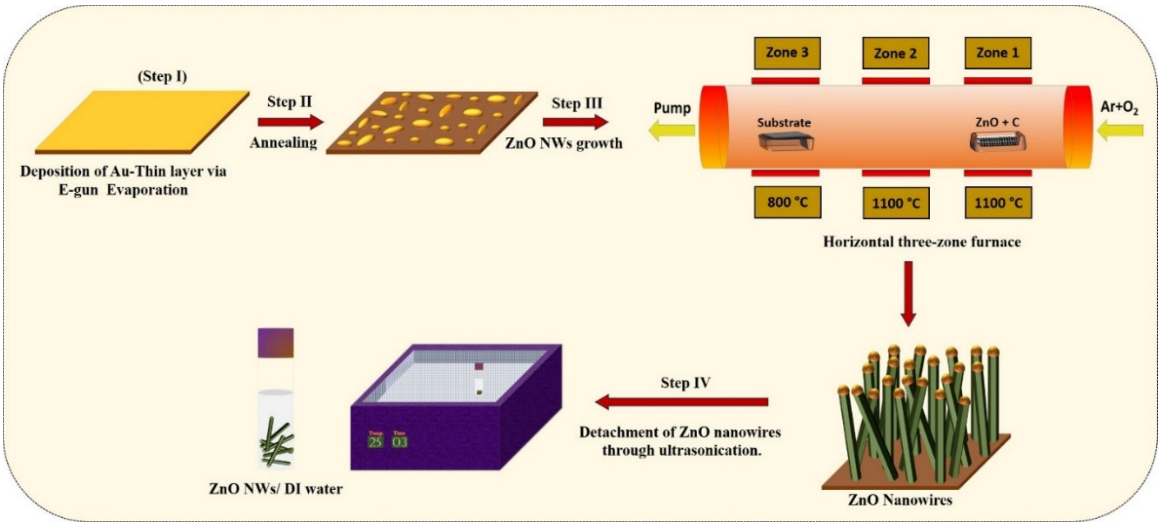

Figure 2. Growth process setup for synthesis of crystalline ZnO nanowires.

3. Setup for photoluminescence and lasing characterizations with a μ-PL system

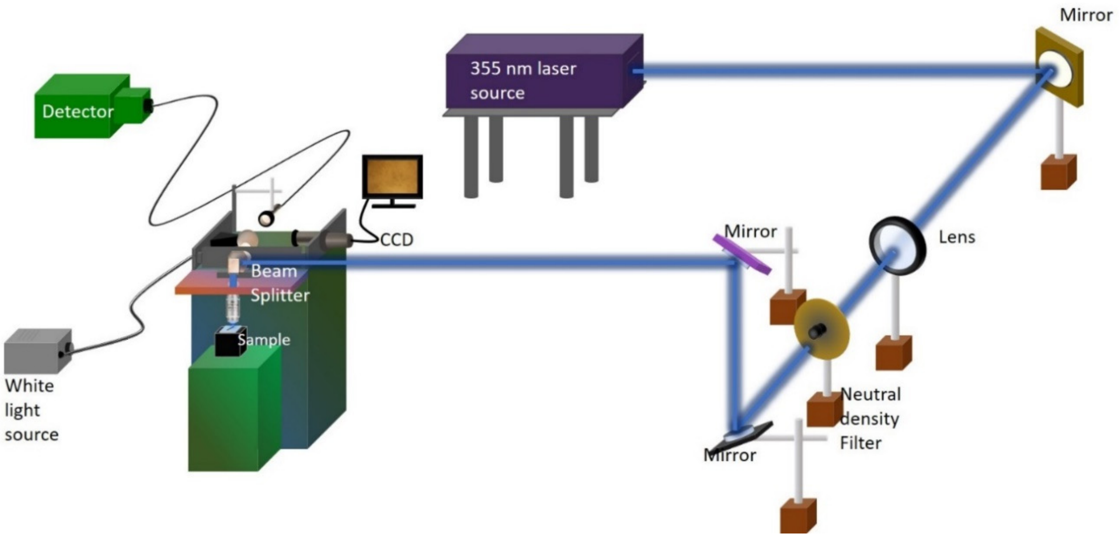

Figure 3. Setup for photoluminescence and lasing characterizations with a μ-PL system.

4. Survey spectra obtained by X-ray photoelectron spectroscopy for WO_3 oxide layer

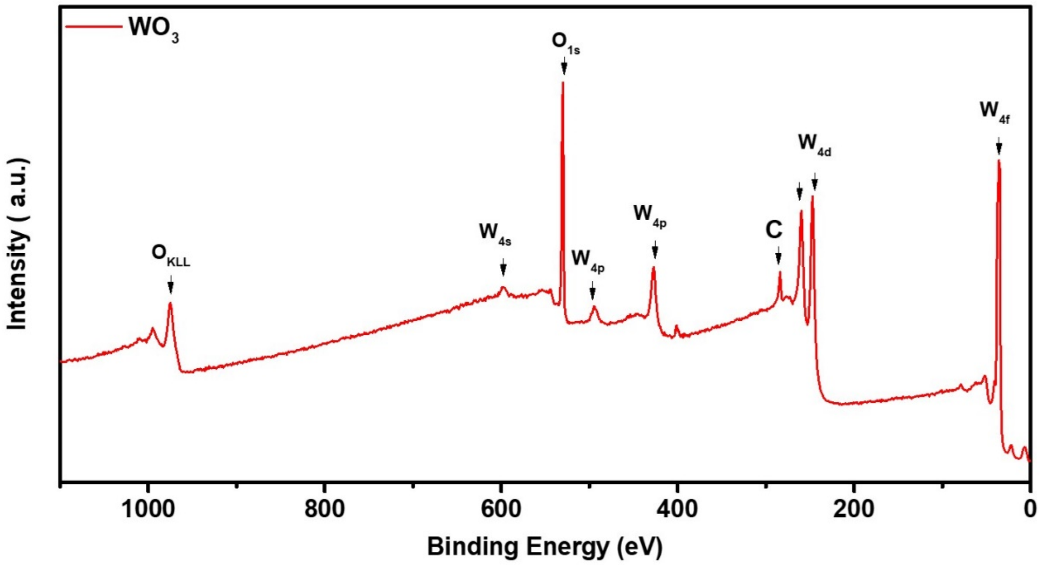

Figure 4. Survey spectra obtained by X-ray photoelectron spectroscopy for WO_3 oxide layer.

5. Dielectric constants obtained by spectroscopic ellipsometry in the wavelength range of 300-1400 nm for different thicknesses of WO_3 oxide layer

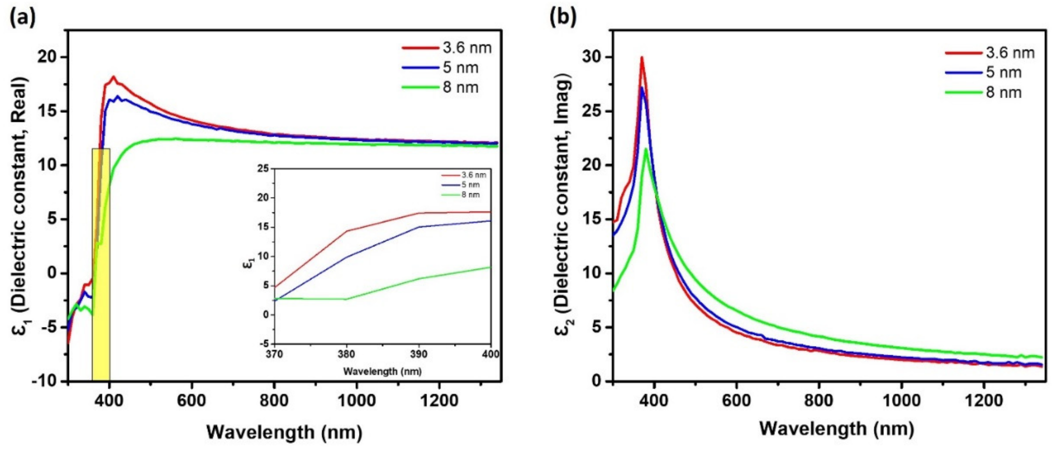

Figure 5. Dielectric constants obtained by spectroscopic ellipsometry in the wavelength range of 300-1400 nm for different thicknesses of WO_3 oxide layer.

6. The simulated effective index for ZnO plasmonic nanolasers from 370–410 nm for different thicknesses of WO_3 layer, obtained from FDTD mode solution method

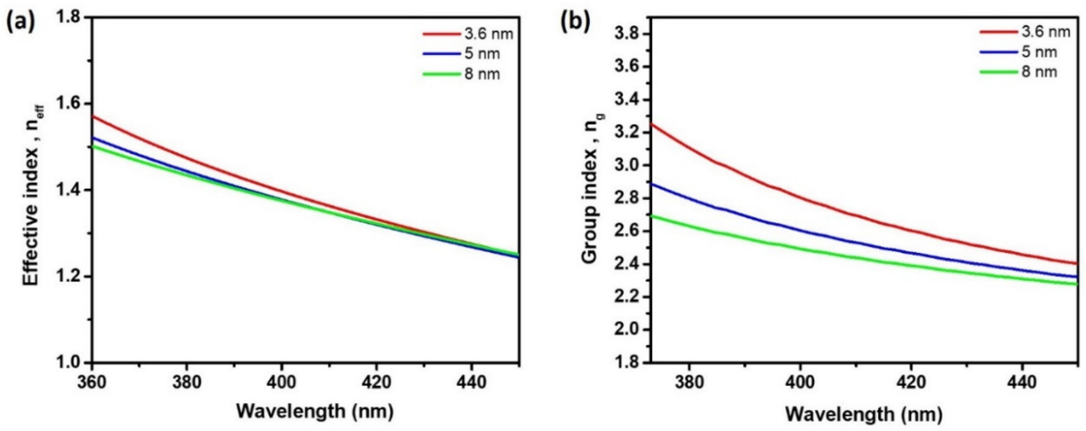

Figure 6. The Simulated (a) effective index and (b) group index for ZnO plasmonic nanolasers from 370-410 nm for different thicknesses of WO_3 layer, obtained from FDTD mode solution method.

7. The AFM profiles of WO_3 layers of different thicknesses

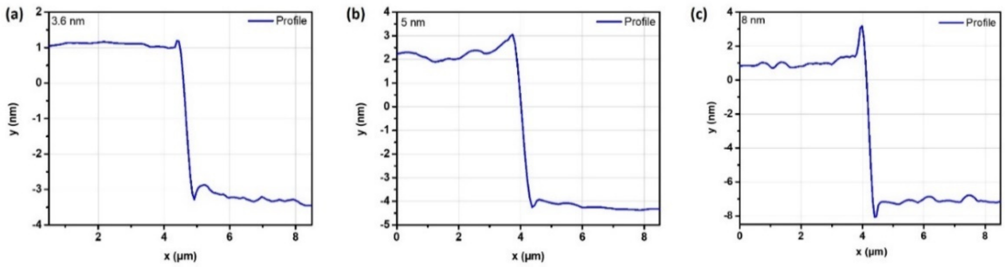

Figure 7. The AFM profiles of WO_3 layers with the thicknesses of **(a)** 3.6 nm, **(b)** 5 nm, and **(c)** 8 nm.

Table 1. Comparison of plasmonic nanowire lasing thresholds with different dielectric thicknesses.

Year	Gain Medium	Metal-Dielectric	Dielectric Thickness	Pump	Temperature	Threshold	References
2020	ZnO	Al-WO_3	3.6 nm<br>5.0 nm<br>8.0 nm	Optical, pulsed	RT[1]	0.79 MW cm^{-2}<br>1.88 MW cm^{-2}<br>2.43 MW cm^{-2}	Current study
2019	ZnO	Al-ALD-Al_2O_3	5 nm<br>10 nm<br>15 nm	Optical, pulsed	RT	6.27 MW cm^{-2}<br>11.1 MW cm^{-2}<br>18.2 MW cm^{-2}	[1]
2016	ZnO	Al-Al_2O_3	5 nm	Optical, pulsed	RT	≈ 100 MW cm^{-2}	[2]
2015	GaAs-AlGaAs	Ag-Non	-	Optical, pulsed	8 K	≈ 1 kW cm^{-2}	[3]
2014	GaN	Al-SiO_2	5 nm	Optical, pulsed	RT	≈ 3.5 MW cm^{-2}	[4]

2012	InGaN	Ag-SiO_2	5 nm	Optical, continuous	78 K	≈ 3.7 kW cm^{-2}	[5]
2009	CdS	Ag-MgF_2	5 nm	Optical, pulsed	10 K	≈ 100 MW cm^{-2}	[6]

[1] RT, Room temperature

References

1. Liao, Y.J.; Cheng, C.W.; Wu, B.H.; Wang, C.Y.; Chen, C.Y.; Gwo, S.; Chen, L.J. Low Threshold Room-Temperature UV Surface Plasmon Polariton Lasers with ZnO Nanowires on Single-crystal Aluminum Films with Al_2O_3 interlayers. *Rsc Adv.* **2019**, *9*, 13600–13607.
2. Chou, Y.H.; Wu, Y.M.; Hong, K.B.; Chou, B.T.; Shih, J.H.; Chung, Y.C.; Chen, P.Y.; Lin, T.R.; Lin, C.C.; Lin, S.D., et al. High-Operation-Temperature Plasmonic Nanolasers on Single-Crystalline Aluminum. *Nano Lett.* **2016**, *16*, 3179–3186.
3. Ho, J.F.; Tatebayashi, J.; Sergent, S.; Fong, C.F.; Iwamoto, S.; Arakawa, Y. Low-Threshold near Infrared GaAs-AlGaAs Core-Shell Nanowire Plasmon Laser. *Acs Photonics* **2015**, *2*, 165–171.
4. Zhang, Q.; Li, G.Y.; Liu, X.F.; Qian, F.; Li, Y.; Sum, T.C.; Lieber, C.M.; Xiong, Q.H. A Room Temperature Low-threshold Ultraviolet Plasmonic Nanolaser. *Nat. Commun.* **2014**, *5*, 1–9.
5. Lu, Y.J.; Kim, J.; Chen, H.Y.; Wu, C.H.; Dabidian, N.; Sanders, C.E.; Wang, C.Y.; Lu, M.Y.; Li, B.H.; Qiu, X.G., et al. Plasmonic Nanolaser Using Epitaxially Grown Silver Film. *Science* **2012**, *337*, 450–453.
6. Oulton, R.F.; Sorger, V.J.; Zentgraf, T.; Ma, R.M.; Gladden, C.; Dai, L.; Bartal, G.; Zhang, X. Plasmon lasers at deep subwavelength scale. *Nature* **2009**, *461*, 629–632.

© 2020 by the authors. Licensee MDPI, Basel, Switzerland. This article is an open access article distributed under the terms and conditions of the Creative Commons Attribution (CC BY) license (http://creativecommons.org/licenses/by/4.0/).
